# Uncommon Infections in Children Suggest Underlying Immunodeficiency: A Case of Infective Endocarditis in a 3-Year-Old Male

**DOI:** 10.1155/2018/9380763

**Published:** 2018-04-01

**Authors:** Aisha Shakoor, Ahmed El-Isa, Elizabeth Kinsella, Ryan Halas, Andrey Leonov

**Affiliations:** ^1^Department of Family Medicine, Western Michigan University, Kalamazoo, MI, USA; ^2^Department of Pediatrics, Western Michigan University, Kalamazoo, MI, USA; ^3^Western Michigan University Homer Stryker School of Medicine, Kalamazoo, MI, USA; ^4^Department of Internal and Pediatric Medicine, Western Michigan University, Kalamazoo, MI, USA; ^5^Department of Pediatric and Adolescent Medicine, Western Michigan University, Kalamazoo, MI, USA

## Abstract

Infective endocarditis (IE) results from bacterial or fungal infection and is associated with significant morbidity and mortality. Several known risk factors exist for endocarditis, and 90% of pediatric cases have an underlying structural or congenital heart disease or prosthetic heart valve. Literature on IE in previously healthy children is relatively sparse, and the pathogenesis and underlying risk factors remain mostly unknown. Our patient was a 3-year-old male with a unique presentation of IE. His lack of structural and congenital risk factors for endocarditis prompted further workup, and labs were consistent with insufficient immunoglobulin, suggesting a primary immunodeficiency (PAD). PAD presents as heightened susceptibility to infections, commonly seen as recurrent pneumonia, meningitis, septic arthritis, and otitis media. Pediatric patients commonly have infections, yet as many as in 1 in 2000 patients have PAD. Our case emphasizes the potential need for further investigation into PAD in a young patient with no known risk factors who develops an uncommon infection such as IE.

## 1. Introduction

IE results from bacterial or fungal infection and is associated with significant morbidity and mortality. Risk factors of IE include congenital abnormality of the heart and prosthetic valves [1, 2]. In patients with native valves and normal cardiac structure, IE has been associated with cardiac surgery secondary to trauma and intravenous drug use [3]. Endocarditis is uncommon in individuals with healthy hearts, approximately 8–10% of cases of IE in pediatric patients occur in the absence of preexisting cardiac disease [4]. Limited literature exists on IE in previously healthy children, and the pathogenesis and underlying risk factors are largely unknown [5].

PAD is a broad category of immunodeficiencies that can result from defects in multiple components of the immune system, including B and/or T cell defects, inadequate or absent antibodies. Various disorders within this category have been identified, including X-linked agammaglobulinemia (XLA), common variable immunodeficiency (CVID), IgA deficiency, and autosomal recessive agammaglobulinemia (ARA). While each of these disorders carries its own unique genetic profile and clinical presentation, patients with PAD often present with recurrent and/or abnormally severe infections. Diagnosis is made based on immunoglobulin levels, flow cytometry and, ultimately, confirmatory genetic testing. PAD patients also have increased risk of both autoimmunity [6] and malignancy [7].

The age of presentation of PAD ranges but can begin to present as early as three months of age following loss of maternal antibody protection. Due to the frequency of infections in young children as their immune systems develop, many physicians may overlook frequent or abnormal infections as normal immature immunity of pediatrics or other benign causes, yet as many as 1 in 2000 children have a PAD [8]. However, delaying diagnosis of PAD is detrimental to health and development. In our case, a 3-year-old male was found to have IE despite having no known risk factors. Our patient illustrates that PAD should be considered in the differential diagnosis of a patient with no underlying risk factors who develops a serious uncommon infection such as endocarditis.

## 2. Case Report

A 3-year-old previously healthy male presented to the emergency department with a fever of unknown origin of approximately four weeks duration. As an outpatient, he had multiple evaluations in the last year and had been diagnosed with pharyngitis followed by otitis media and received full courses of amoxicillin and azithromycin. Despite antibiotics, he continued to have daily fevers up to 102.5 F. He also had complaints of weight loss (7 lbs), fatigue, nonproductive cough, and constipation. Vital signs at presentation included a fever of 101.7 F, heart rate of 135, and a respiratory rate of 20.

Physical exam revealed dry mucous membranes, posterior pharynx erythema without tonsillar exudates, posterior cervical lymphadenopathy, normal cardiovascular and respiratory exams, mild abdominal distention without organomegaly or significant tenderness, and delayed capillary refill of four seconds. Other exam findings were within normal limits.

**Figure 1:**
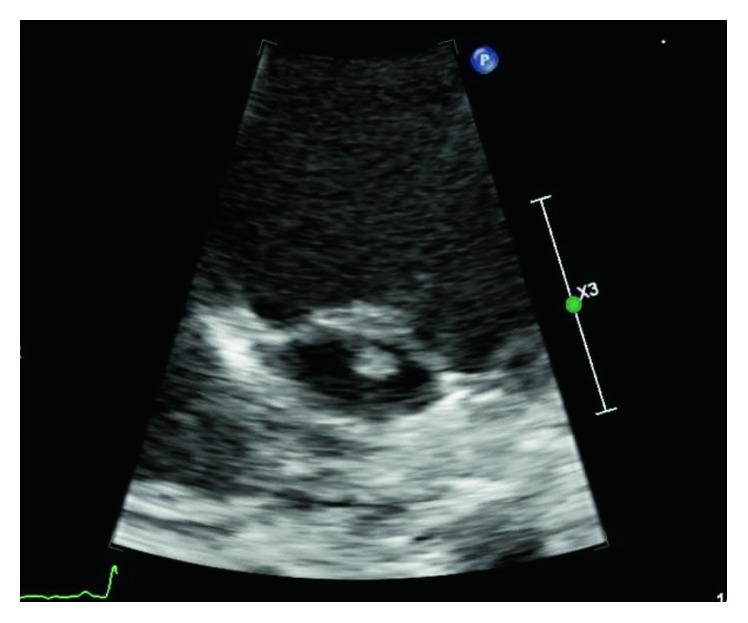
Small 3 × 5 mm vegetation on atrial aspect of the mitral valve as visualized on echocardiogram Apical four chamber view with apex up and posterior angulation.

Blood culture was positive for *Enterococcus faecalis*, prompting transthoracic echocardiogram which was positive for a 3 × 5 mm vegetation on the anterior mitral valve leaflet confirming endocarditis (Figure 1). The patient was started on intravenous ampicillin, which was terminated 14 days into therapy due to a suspected ampicillin-induced neutropenia (ANC 100 cells/mm^3^). He was then transitioned to vancomycin and gentamicin for a total antibiotic duration of four weeks.

After completion of his four-week course of antibiotics, the patient continued to develop recurrent fevers, and further infectious disease showed a PCR positive for *Clostridium difficile*, which was treated with metronidazole. Given his history of endocarditis with no identifiable risk factors, an immunological workup was perused. The workup was consistent with low IgG, IgA, and IgM levels (Table 1).

Following the immunology workup, the patients Bruton tyrosine kinase (Btk) gene was sequenced to evaluate XLA. The identified gene was c. 1178-3T > G, a variant of uncertain significance in the Btk gene, presumed to be the cause of his agammaglobulinemia. Further immune assessment of the B cell subsets as seen in Table 2 showed an absence of CD19+ B cells. The intracellular Btk protein expression was decreased in monocytes (MFI = 2.06) relative to experimental control (MFI = 7.99). This result appears to be suggestive of a diagnosis of XLA.

Since the diagnosis, he has been receiving regularly scheduled intravenous immunoglobulin (IVIG) infusions and remains symptom free for the last four months.

## 3. Discussion

IE is rare in the pediatric population and carries a high risk of morbidity and mortality. The incidence has been reported at a rate of 0.34 to 0.64 cases per 100,000 per year [5]. Majority of cases result from a structural defect or congenital heart disease [2]. Approximately 10% of cases have no underlying heart disease, and the cause is largely unknown [4]. Our case is of a 3-year-old who presented with endocarditis without any identifiable risk factors who was found to have PAD upon further workup. Since pediatric patients commonly have infections, PAD is commonly overlooked. In the cases of abnormal infections the cause must be identified, and PAD should be at the top of that differential.

Majority of patients with PAD are diagnosed when they develop a severe infection secondary to their lack of protective immunoglobin [9]. Infections which are commonly reported include recurrent pneumonia, empyema, sinusitis, recurrent otitis, sepsis, recurrent meningitis, or septic arthritis [10–12]. In those without protective B cells, bacterial infections typically begin three months after birth when maternal IgG is reduced below the protective level and most patients are diagnosed prior to the age of five [12]. Many of these infections are not severe and are also often seen in immunocompetent children. The occurrence of serious infections such as IE however is rare and an important clue to prompt further workup to identify any underlying PAD.

There are several types of PAD. One example is the XLA or Bruton's agammaglobulinemia which is a rare genetic disorder with a prevalence of 1 in every 19,000 male births in the United States [13]. B cell formation is attenuated due to a mutation in the *Btk* gene which alters the Btk protein involved in B cell differentiation [14, 15]. The *Btk* gene was identified in 1993, and since then numerous gene mutation sites have been identified [7]. Our patient has a variant Btk mutation that has not been previously reported in literature. The clinical picture of our patient is consistent with a lack of B cell differentiation causing a lack of immunoglobulin and subsequently allowing recurring and serious infections; therefore, it is plausible that the mutation can be causative. It is important to note however that this variant gene mutation is of unknown significance and further investigation into its relevance for agammaglobulinemia is ongoing.

In conclusion, endocarditis is a serious infection which is uncommon in the healthy pediatric population with no known risk factors. PAD is often overlooked as immunocompetent children often present with common infections such as otitis media and pneumonia. Our case emphasizes that pediatric patients presenting with more serious and uncommon infections such as endocarditis should be carefully evaluated for underlying cause. Our case emphasizes that PAD should be at the top of the differential in such patients as delay in diagnosis and treatment can lead to increased morbidity and mortality.

## Figures and Tables

**Table 1:** Immune workup showed the following labs.

Immunoglobin	Level	Reference range
IgG	<30	331–1090 mg/dL
IgA	<5	13–157 mg/dL
IgM	6	41–190 mg/dL

**Table 2:** Immune assessment B cell subsets.

Lymphocyte subsets	Level	Reference range
% CD19 (B cells)	0%	13–39%
CD19 (B cells)	0 cells/mcL	370–2306 cells/mcL
